# Modified Polyethylene Foams for Insulation Systems

**DOI:** 10.3390/polym15204104

**Published:** 2023-10-16

**Authors:** Sabu Thomas, Karapet Armenovich Ter-Zakaryan, Aleksey Dmitrievich Zhukov, Igor’ Vyacheslavovich Bessonov

**Affiliations:** 1 School of Energy Materials, Mahatma Gandhi University, Kottayam 686560, Kerala, India; 2 OOO TEPOFOL, Moscow 105318, Russia; karo73@mail.ru; 3 Department of Building Materials Science, National Research Moscow State University of Civil Engineering (NRU MGSU), Moscow 129337, Russia; lj211@yandex.ru; 4 Research Institute of Construction Physics, Russian Academy of Architecture and Construction Sciences, Moscow 127238, Russia; bessonoviv@mail.ru

**Keywords:** polyethylene foam, insulating shell, flame retardant, modified clay, oxygen index, properties prediction

## Abstract

Effective insulation of buildings and other industrial objects requires the use of materials and system solutions that ensure maximum uniformity and density of insulation shells. The study focuses on the development of insulation systems based on expanded polyethylene and, in particular, on the development of modified polyethylene with reduced flammability containing a flame-retardant modified montmorillonite clay, which does not hinder gas formation, and silicate nanofillers in layered construction. Active experiments based on mathematical design methods allowed us to establish an analytical relationship between flame-retardant and modifier consumption and extruder pressure and response functions: average density of polyethylene foam and flammability criterion. The flammability criterion was taken as the oxygen index of the modified polyethylene foam. A foaming agent masterbatch was used as the flame retardant. Analytical optimization of mathematical models obtained as a result of active experiments allowed us to determine the optimal flame-retardant consumption, which was 3.7–3.8% of the polymer mass. Optimised systems for average density and oxygen index of flammability of modified polyethylene were obtained. A nomogram for predicting the material properties and selecting the composition, and an algorithm for a computer program for evaluating the properties of modified polyethylene foam as a function of the values of various factors, were developed. Taking into account the possible expansion of the scope of application of rolled polyethylene foam and seamless insulation shells based on it, possible solutions for insulation systems were studied using the program THERM, and a combined insulation system was adopted.

## 1. Introduction

Building insulation systems today are necessary constructive additions to any building structure: facade, roof, and conditions of these spaces. Accordingly, polyethylene foam has several advantages, such as low thermal conductivity, low vapour and water permeability and the ability to form seamless joints on the surfaces to be insulated. Construction practice shows that the fewer connections between the individual elements of the insulation shell, the higher the thermal resistance. For structures in regions with milder climates, maintaining comfortable conditions remains a priority. For technological facilities and storage facilities for agricultural products, the main issue is to maintain the required microclimate (in terms of air temperature and humidity) in the insulated rooms. There are also systems designed to maintain negative temperatures in insulated spaces: retaining cold, keeping snow in the off-season, and keeping the ground in a permafrost state [1,2,3].

The basis of insulation systems is thermal, hydro, and vapour barrier materials that form protective shells. The hydro and vapour barriers are usually made of rolled polymeric materials based on polyethylene, modified bitumen, and membranes of various types. Products based on mineral fibres, expanded polystyrene and polyurethane, as well as gas-filled polyolefins are used as effective insulation. Products in the form of mats or rolls are used, as well as spray insulation, but in most cases, board products are used [4,5,6].

The use of slab products in the insulation shell (based on glass or rock wool or extruded polystyrene foam) is very widespread, although they have a number of peculiarities [7,8,9]. First, at the contact surfaces between the panels, it is almost impossible to achieve a snug fit of these panels; second, similar effects occur at the contact surfaces of the slab thermal insulation and the elements of the supporting structure; third, the mechanical fastening of the panels requires the use of dowels and other fasteners. All these features indicate the formation of areas of increased heat transfer due to both conductive processes and convection (suction of cold air through leaks). Areas of increased heat transfer (cold bridges) increase the thermal inhomogeneity of insulation layers and decrease their thermal resistance [10,11,12].

We also point out the influence of the human factor caused by mistakes in the design or installation of insulation shells. In order to limit heat losses to certain limits, special control methods are used, such as thermal imaging of objects, thermal imaging of rooms, and instrumental methods for determining heat flow through insulated building structures.

The problem of heat loss is exacerbated during severe winters or during construction in the climatic conditions of the Arctic, when any “cold bridge” can lead to significant heat loss and additional loads on structural elements, including load-bearing elements, which in turn can affect their reliability and integrity [13,14,15]. Construction practice shows that the fewer joints there are between the individual elements of the insulation shell, the more uniform it is and, consequently, the greater the thermal resistance at the surface of the insulated surfaces.

Such shells (with a minimal number of seams or seamless) can be made by spraying (polyurethane foam) or by using rolled materials (polyethylene foam). The use of polyurethane foam in building insulation systems is limited by the following factors. First, when polyurethane foam is used on a construction site, strict health safety requirements must be met (the components are usually toxic). Second, the temperature of the environment in which the insulation is applied must be at least 5 °C, which makes the use of this method seasonal. Third, the material is adhesively attached to the substrate, which means that if the coefficients of thermal expansion (compression) do not match, the insulation layer may peel off and the insulation system may be destroyed.

**Figure 1:**
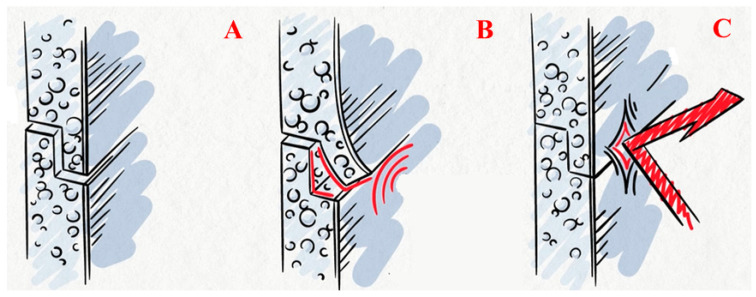
Scheme of forming a seamless insulating shell: (**A**) interlock; (**B**) hot-air welding (construction heater) along the seam; (**C**) formation of a seamless insulating shell.

When rolled polyethylene foam is used in insulation systems, it is mechanically attached to the surface and then forms a seamless joint (Figure 1).

The technology for forming a seamless joint with subsequent fusion of the contact surfaces is realised with polyethylene foam, which is melted over the contact surfaces and subsequently solidifies when the heating source is removed [16,17]. These materials belong to the group of foamed plastics and have both the general advantages of this group (low thermal conductivity, vapour and water resistance) and a common feature—combustibility [18,19].

Flammability is a criterion for the fire hazard of a structure or system and is expressed by the ability of a material to ignite, sustain and continue combustion. This process depends on several variables: ignition temperature, auto-ignition temperature, burnout rate, fire spread rate, heat release intensity, and toxicity. This process is also affected by factors such as atmospheric composition, air density, and oxygen index.

The oxygen flammability index (%) is a characteristic used to evaluate the flammability of polymeric materials. The index indicates the minimum oxygen content in the nitrogen–oxygen mixture at which the polymer material can still ignite. The higher the oxygen content in the nitrogen–oxygen mixture, the higher the oxygen flammability index and the lower the flammability of the material. The oxygen index indicates the minimum oxygen content at which combustion of the material is possible.

Reducing the flammability of foamed plastics is a more than urgent task and is mainly implemented with the help of technological methods. Foams based on polyolefins (polyethylene or polypropylene) are foamed with a gas dissolved in the polymer under pressure in an extruder.

The core of the method is to melt the polymer and introduce modifying additives into its composition. In the extruder, the polymer is in a viscous state and becomes saturated with gas at elevated pressure. With the subsequent release of pressure at the exit of the extruder (in the mould) and a rise in temperature, the solubility of the gas decreases sharply and it begins to be released in the form of gas bubbles. The resulting cell structure is fixed by “physical” or “chemical” methods, depending on the structure of the resulting polymer. For thermoplastic polymers, cooling the foam is sufficient to fix the structure.

The flammability of foamed polyolefins is reduced, on the one hand, by introducing flame retardants into the polymer and, on the other hand, by foaming with gasses that do not support combustion (e.g., CO_2_), as well as by producing a polymer with a fire-extinguishing component in its structure and by producing composites based on polyethylene [20,21,22]. The main requirements for polymer composites with enhanced combustion resistance are low smoke emission, absence of toxic gasses and burning melt droplets during combustion of the products made from them, while maintaining a high level of physical and mechanical properties [23,24].

Reducing the flammability of the polymer matrix by using layered silicates, namely talc, clay (including montmorillonite), mica, aluminium silicate, and silicon silicate, has been studied both abroad and in Russia. Modified clay (organoclay) has a number of advantages over unfilled clay, including the ability to be well dispersed in the polymer matrix. Ammonium alkyl or phosphonium alkyl, pentaerythritol or maleic anhydride can be used as modifiers. The optimum filler concentration to achieve a high level of physical and mechanical properties is 2% organoclay [25,26].

During the pyrolysis of layered silicates (montmorillonite), the interlayer water contained in the clay minerals (montmorillonite) evaporates and the resulting gaseous products within the clay layers block the propagation of the flame. The reactions of pyrolysis and evaporation of water are endothermic and contribute to lowering the combustion temperature. The water that evaporates between the packages contributes to the formation of a fire barrier.

The formation of gaseous products (including water vapour) leads to the swelling of clay minerals (montmorillonite) and the formation of rigid foam, which plays the role of an insulator against heat and oxygen. Moreover, some of the gaseous products in the clay layers are capable of producing coke (Frank–Rabinovich cell effect).

**Figure 2:**
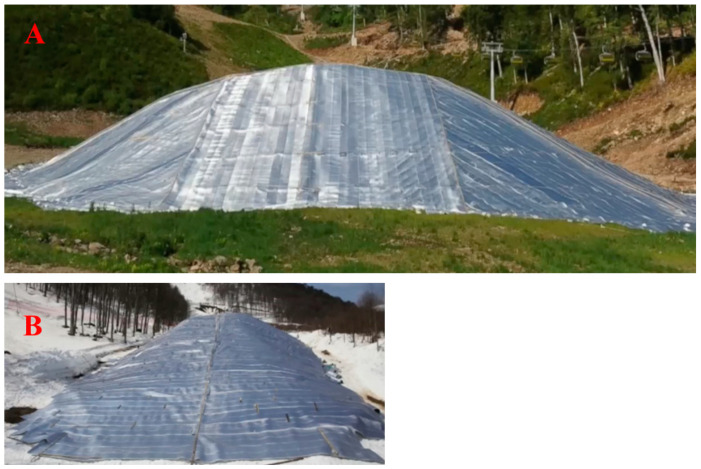
Preservation of snow in the off-season: (**A**) laying snow for storage in early spring; (**B**) storage of snow in summer.

Insulating shells based on foamed polyethylene are used to produce thermal insulation and sealing systems for framed and frameless building structures, to insulate refrigerators and other equipment for cold protection, and especially for protection against snow in the off-season (Figure 2). Most in demand are systems for low-rise buildings, storage and utility rooms, and agricultural product storage rooms. The use of polyethylene foam shells enables heat savings, the creation of a comfortable microclimate in the rooms and the maintenance of the temperature and humidity conditions required for the technologies in the rooms.

**Figure 3:**
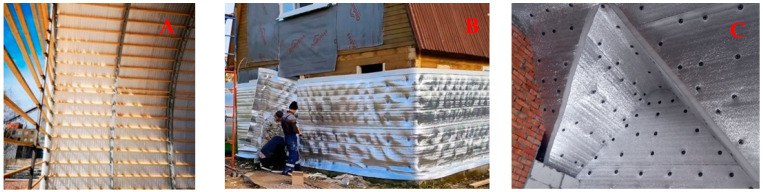
Rolled polyethylene foam in the insulation of building structures: (**A**) insulation of a frameless hangar; (**B**) insulation of the walls of a low-rise building; (**C**) attic insulation.

A special feature of the arrangement of insulation shells in the insulation of walls and pitched roofs of buildings and structures is that the insulation material can be arranged with a foil or metallized surface in systems protected from external influences, e.g., in a floating floor structure (Figure 3), or in situations where contact with the environment cannot be completely avoided. This results in additional requirements for the fire safety of enclosing structures. As a rule, such insulation systems are used for category V buildings, but the question arises as to how the flammability of insulation materials can be reduced.

When insulating building profile objects, seamless insulation shells can be formed by using rolled polyethylene foam and installing these systems based on TEPOFOL technology. Products on insulation bases or in frame systems are mechanically fixed. The expansion of the scope of application of polyethylene foam is directly related to the study of the possibility of reducing its flammability. The best result can be achieved by implementing a comprehensive solution.

Reducing the flammability of foamed polyolefins is carried out, first, by introducing flame retardants into the polymer and, second, by foaming with gasses that do not support combustion (e.g., CO_2_), as well as by creating a polymer with a fire-extinguishing component in its structure and by creating composites based on polyethylene [20,21,22]. The main requirements for polymer composites with enhanced combustion resistance are low smoke emission, absence of toxic gasses and burning melt droplets during combustion of products made from them, while maintaining a high level of physical and mechanical properties [23,24].

The purpose of the research presented in the article was to improve insulation systems based on polyethylene foam, and, in particular, to develop a modified low-flammability polyethylene containing a flame retardant that does not prevent gas formation and layered silicate nanostructured fillers: modified montmorillonite clays.

## 2. Materials and Methods

**Figure 4:**
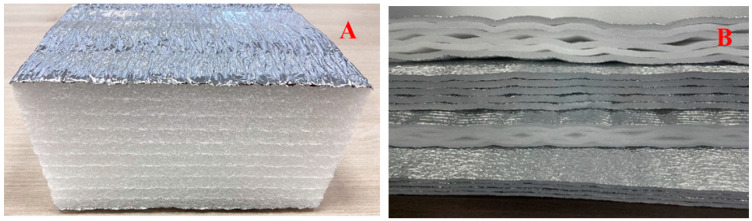
Products made of polyethylene foam: (**A**) layered products; (**B**) products of the AirLayer line.

The formation of insulating shells with the implementation of the technology of seamless connection involves the use of two types of roll products based on polyethylene foam (with or without a heat-reflecting coating). When forming seamless insulating shells, the following products are used. Firstly, layered products with a thickness of 10 to 200 mm (and more if necessary) with layers welded over the entire contact plane (Figure 4A). Secondly, materials of the AirLayer line (RF patent No. 199048) [17]. These are heat-insulating multilayer materials containing flat layers of foamed polyethylene (polypropylene or rubber) connected by spot welding (Figure 4B).

Depending on the conditions of use, the width of polyethylene foam rolls can be up to 2 m, and the length of the material is made to the customer’s size. An edge is formed on the sides of the rolls, allowing the formation of a lock connection. In accordance with TEPOFOL technologies, individual sheets are joined into a lock and welded with a building hot air gun, thereby forming a seamless insulating shell (RF patent No. 2645190) [16].

To confirm the possibility of using polyethylene foam under various operating conditions, studies were conducted at the Scientific Research of the Moscow State University of Construction (NRU MGSU) and the Research Institute of Building Physics (NIISF), as well as at the production base and research laboratory TEPOFOL LLC (Russia, Moscow) and at the laboratories of Mahatma Gandhi University (India).

The test to evaluate the flammability of modified polyethylene foam was carried out according to the methodology of mathematical design based on a complete three-factor plan, followed by the testing of statistical hypotheses, analytical optimization and graphical interpretation of the results. Maleic anhydrite was used as a modifier. A flame retardant and an exothermic nucleating agent, Foaming Agent Masterbatch 19070029 with a decomposition temperature of 170–190 °C, were used in the studies. Foaming was achieved by the introduction of carbon dioxide. The test conditions are shown in Table 1.

As response functions, the following are accepted: the average density of products made of polyethylene foam (Y_1_, kg/m^3^) and the value of the oxygen flammability index of the modified polyethylene foam (Y_2_, %). As an optimisation parameter, the oxygen index is taken as a response characterizing the conditions for the ignition of polyethylene foam.

The determination of the oxygen index was carried out according to the standard method specified in the set of rules “Fire and explosion hazards of substances and materials”, which is valid in Russia and the countries of the Commonwealth. 

The oxygen flammability index of polyethylene was known (17.4%); therefore, given that the material burns in air, the tests began with 16% oxygen mixed with nitrogen. The oxygen index in percent was calculated by Formula (1):

(1) $$I_{o}=\frac{V_{o}}{\left(V_{o}/V_{a}\right)}\times 100$$

where V_o_—volume flow rate of oxygen, L/min or cm^3^/s; and V_a_ is the volume flow rate of nitrogen, L/min or cm^3^/s.

The determination of the average density of the products, as well as other properties of polyethylene foam, was carried out according to the methods specified in the national standard (update EN 14313:2009 [27]) “Factory-made heat-insulating polyethylene foam products”.

Visualization of temperature fields and heat flow distribution in insulation systems was performed using the computer program THERM. The program allows creating models of two-dimensional heat transfer in building elements. Heat transfer analysis with the program allows evaluating the energy efficiency of the construction and the distribution of local temperatures, solving problems with condensation, humidity of the building material and its air tightness.

## 3. Results and Discussion

The experiment (whose conditions are shown in Table 1) was performed on the basis of a three-factorial experimental matrix constructed on the basis of a D-optimal rotation plan. At each point of the plan, the experiments were performed at least five times. To reduce the possible influence of extraneous factors, the order of experiments was randomized. The “scatter” of the results for each set of experiments was evaluated using the Cochran criterion.

The processing of the experimental results with the program Statistika allowed us to create a model (algebraic polynomial or function of three variables) relating the oxygen index and the average density of the polyethylene foam to the changes in the variable factors. The significance of the coefficients of the basic polynomials was evaluated using confidence intervals (Δb) calculated with the Student *t*-test. The adequacy of the obtained models was checked using the Fisher criterion. As a result, the following basic mathematical models were determined:

- for average density (with a confidence interval Δb = 1.4):
  Y_1_ = 28.2 − 4.2X_1_ + 0.4X_2_ − 2.7X_3_ + 1.4X_1_X_3_ − 0.8X_2_^2^ (2)
- for the oxygen index (with a confidence interval Δb = 1.2):
  Y_2_ = 25.6 − 1.8X_1_ + 6.4X_2_ − 1.6X_3_ − 1.0X_1_X_3_ (3)

By analysing the magnitudes and signs of the coefficients of the regression equations, it is possible to determine the degree and direction of influence of each of the variable factors on the average density and fire properties of polyethylene foam.

The average density of polyethylene foam (Y_1_, kg/m^3^) is most dependent on the consumption of the modifying additive; moreover, the density of polyethylene foam decreases as the consumption of the additive increases (the coefficient at “X_1_” is equal to “−5.2”). This is understandable from the point of view of the mechanism of action of maleic anhydride, which has a plasticising effect on the melt under the conditions of polymer melting in an extruder. The consumption of flame retardant is insignificant but increases the average density of polyethylene foam. In addition, the density may also decrease at high flow rates (the coefficients at “X_2_” and equal to “X_22_” are “1.4” and “−0.8”, respectively), which is due to a change in surface tension in the polymer system with the introduced flame retardant. It was found that the combined effect of the flow rate of the modifier and the pressure in the extruder leads to a slight increase in the average density (the coefficient at “X_1_X_3_” is equal to “−1.4”), which is due to the possible partial breakthrough of the blowing agent during foaming in the last section of the extrusion.

The oxygen flammability index of the modified polyethylene foam (Y_2_, %) as a characteristic of the material’s ability to perceive fire loads depends most on the flame retardant content (the coefficient at “X_2_” is equal to “6.4”); the influence of other factors is shown to a lesser extent (coefficients at “X_1_, X_3_, X_1_X_3_” equal to “−1.8”, “−1.6” and “−1.0”, respectively) and is due to the degree of porosity (foaming) of the polymer matrix. Analytical optimization according to the methodology of NRU MGSU includes the following steps: determination (by analytical methods) of the value of the optimal flame-retardant consumption, and determination of the optimised dependencies of the average density of the modified polyethylene foam and its oxygen index.

(1) Determination of the optimal consumption of flame retardant:
  ∂Y1∂X2=1.4−1.6X2=0→X2=1.41.6=0.88

Or in the natural form (taking into account the data of Table 1): Pa = 3 + 1 × (0.88) = 3.7 … 3.8%.

(2) Obtaining optimised algebraic models, we substitute the optimal flame-retardant consumption in coded values into Equations (2) and (3):
  - for medium density:
    Y_1_ = 28.2 − 4.2X_1_ + 1.4(0.88) − 2.7X_3_ − 1.4X_1_X_3_ − 0.8(0.88)^2^
  - for the oxygen index (with a confidence interval Δb = 1.2):
    y_2_ = 25.6 − 1.8X_1_ + 6.4(0.88) − 1.6X_3_ − 1.0X_1_X_3_
  - for medium density:
    Y_1_ = 28.8 − 4.2X_1_ − 2.7X_3_ − 1.4X_1_X_3_ (4)
  - for the oxygen index:
    Y_2_ = 31.2 − 1.8X_1_ − 1.6X_3_ − 1.0X_1_X_3_ (5)

**Figure 5:**
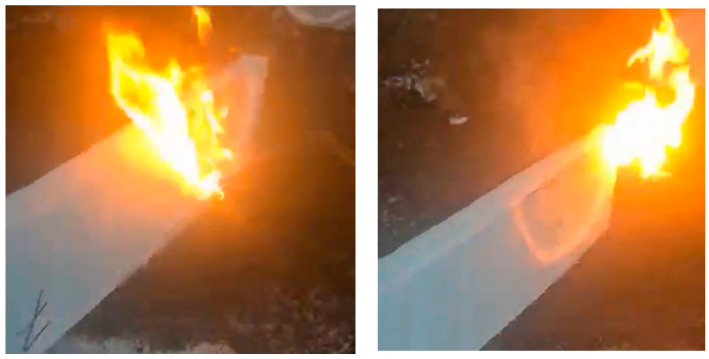
The nature of combustion of a sample of polyethylene foam in contact with an open flame of a burner.

The combustion conditions of the modified polyethylene foam in contact with the burner flame are shown in Figure 5. At optimum flame-retardant consumption (3.7 … 3.8%), the flame extinguishes automatically in air for 20–40 s, and there is no drop formation of the melt, which burns on contact with the burner flame.

The open test (Figure 5) included 24 samples. The average density of the polyethylene foam is 30–32 kg/m^3^; the cell size is 0.5–0.8 mm. Flame source: gas burner (diameter 80 mm, flame temperature in the burner 1000 °C, surface temperature 400 °C). Duration of contact 20 min.

**Figure 6:**
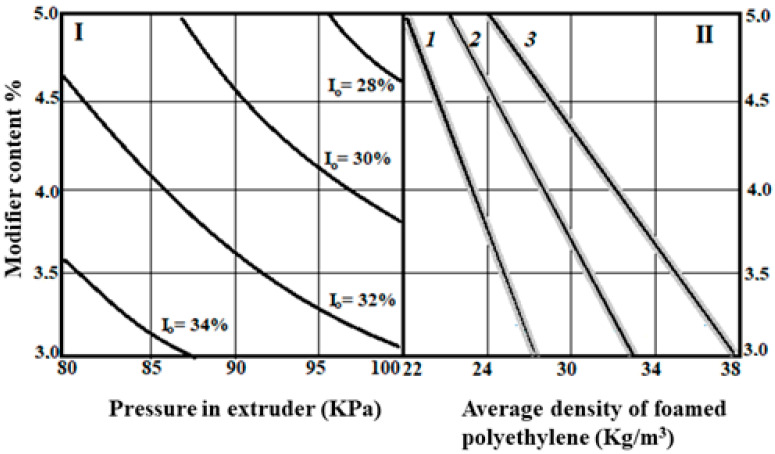
Nomogram of modified polyethylene foam properties: Pressure in the extruder: 1—80 KPa; 2—90 KPa; 3—100 KPa; I—the average value of the result (mathematical expectation); II—deviation of the result from the mean value (dispersion of the result).

The nomogram for predicting the properties of polyethylene foam and determining the characteristics of its composition and technological parameters (taking into account the data in Table 1) was created based on the results of a graphical interpretation of algebraic polynomials (4 and 5). The nomogram can be used to solve direct (prediction) and inverse (selection of parameters) problems of mathematical modelling (Figure 6). 

Since the processing of the experimental results is statistical in nature, which means that due to the statistical errors of the experiment there is constantly a probability factor, in addition to the curve describing the average value, the variance of the result is inserted into the diagrams (sector II), i.e., the deviation of this result from the average value.

The nomogram consists of two sectors. Sector I establishes a graphical relationship between the oxygen index of the material and variable factors. Sector II inserts the average density of polyethylene foam and the same variable factors. With the help of this nomogram, it is possible to solve both the problem of prediction and the problem of composition selection.

After the results are obtained analytically (according to Equations (4) and (5) or according to the nomogram in Figure 6), the possible discrepancy between the density and oxygen index values predicted by the model and the actual values must be checked in repeated series of the active experiment. The results of a comparative analysis with optimum flame-retardant consumption (3.7 … 3.8%) are shown in Table 2.

The deviation between the calculated and experimental values for the average density is not more than 5.2% and for the oxygen flammability index of the modified polyethylene foam it is 4.8%. These indicators agree well with the test results in the evaluation of the properties of building materials. Taking into account the fact that the oxygen flammability index was used as a parameter for optimizing the results, the best indicators are compositions 4, 5 and 6, which are characterized by the consumption of the modifier and approach the lower level of variation of this factor. The obtained results agree well with the test data mentioned in the introduction, according to which an excessive consumption of modifier of more than 4% is not desirable.

**Figure 7:**
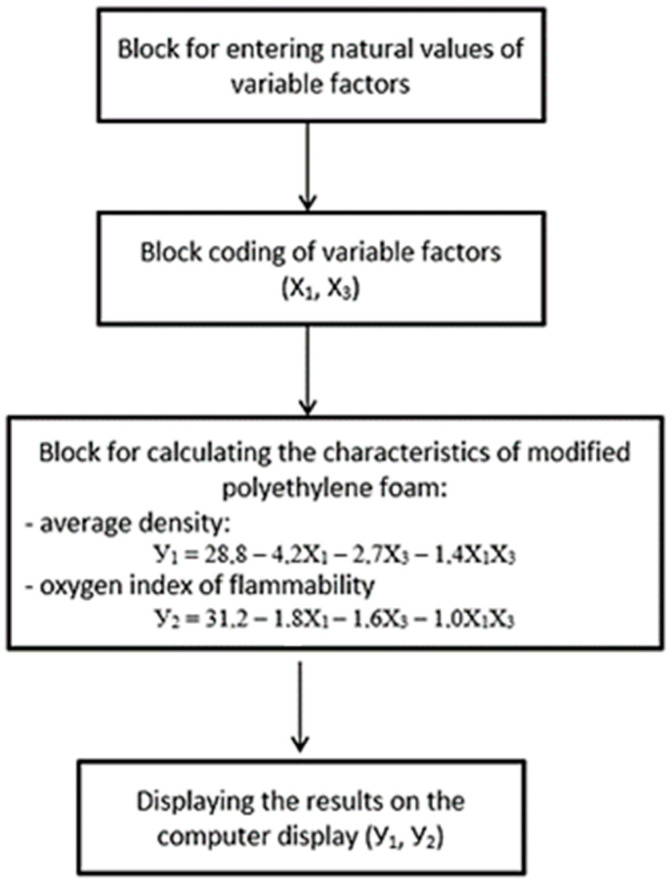
Algorithm of a computer program for predicting the properties of modified polyethylene foam at an optimal flame-retardant consumption of 3.7 … 3.8%.

The solution of the prognostic problem of the evaluation of the properties of modified polyethylene foam depending on the given values of the variable factors can be implemented with the help of a computer program, the algorithm of which is shown in Figure 7.

The algorithm is based on sequential execution of operations: introduction of the factors, coding of the factors; calculation of the values of the response functions and display of the results on a computer screen and printout. The coding block, based on the data in Table 1, allows the transition from the natural values of the factors to the reduced coded interval [−1, +1]. 

The transition to the coded form and back to the natural form is carried out according to the following formula:

$$\mathrm{Calculation}\;\mathrm{formula}\;\mathrm{for}\;\mathrm{coding}:\;X_{i}=\frac{X_{cpi}-X_{Hi}}{\Delta X_{i}}$$

$$\mathrm{Calculation}\;\mathrm{formula}\;\mathrm{for}\;\mathrm{decoding}:\;X_{Hi}=X_{cpi}+\Delta X_{i}$$

where: X_Hi_—natural value of the i-th factor; X_pi_—average value of the i-th factor ΔX_i_—variation interval of the i-th factor; X_i_ is the coded value of the i-th factor.

The calculation block of the algorithm contains optimised mathematical polynomials relating the average density of the modified polyethylene foam and its oxygen flammability index to the modifier consumption and the pressure generated in the extruder (optimised mathematical models 4 and 5). At the same time, the optimal values for flame-retardant consumption (3.7 … 3.8%) do not change. The “Data output” block allows printing the results of compressive strength and average density of the composite binder in natural units or tracking them on the computer.

Reducing the flammability of polyethylene foam makes it possible to significantly expand the scope of application of this material and increase the level of safety of structures, both in terms of potential fire load and in terms of increasing reliability and durability. The production of modified polyethylene foam, which has the ability to self-extinguish when exposed to fire again, allows the use of insulation shells on this basis in buildings below category V and in structures where polyethylene foam is used directly with an open surface. The possibilities for the use of rolled polyethylene foam in construction in arctic conditions are increasing. Under these conditions, a jointless insulation system is particularly effective (see Figure 1).

Construction in northern latitudes, in the Arctic and in permafrost conditions is complicated both by environmental parameters and logistical problems, and by the peculiarities of the condition of isolated objects. If the material belongs to the group of dangerously flammable substances, even transportation and storage in warehouses involves a certain risk and possible losses. If the material is flame-retardant and has the ability to self-extinguish, the logistical risks are significantly reduced.

It should be noted that slight leaks in insulation shells, which are not critical at mid-southern latitudes, can become a problem at sub-zero temperatures below 40 degrees and significant wind speeds that can exceed 20 m/s, resulting in significant heat loss from insulated spaces.

Under these conditions, the material’s performance characteristics, its direct fire properties, and its ability to produce seamless shells are equally important. Modified polyethylene foam in roll form can be considered a priority material under such service conditions. Rolled modified polyethylene foam as a base for seamless insulation boards can be used to make an insulation shell for buildings on pile foundations and as a structural element to protect permafrost soil from thawing.

Materials for thermal insulation systems for buildings and structures in northern latitudes should have not only low thermal conductivity, but also high stability of operation and properties under difficult conditions of use. Also important are the vapour and water permeability of such materials, as well as resistance in aggressive environments, including contact with groundwater. These requirements are met by rolls of polyethylene foam.

**Figure 8:**
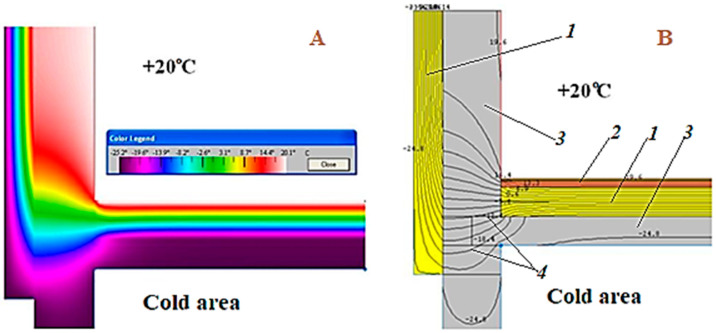
The structure of the formation of the temperature field in a structure that is not isolated above the ventilated space: (**A**) visualization of the temperature field; (**B**) graphical interpretation of the temperature distribution (thermal insulation is laid along the base of the ceiling and along the surface of the vertical walls): 1—slab thermal insulation (XPS or PIR); 2—chrysotile cement sheet; 3—load-bearing structural elements; 4—butt joints between slabs.

To get an idea of how temperatures and temperature fields are distributed, they can be visualized. The computer program THERM was used to simulate the conditions for modelling two-dimensional heat transfer in the building envelope of a pile structure (Figure 8). The construction of such a building takes place on frozen ground.

In polar winters, when the temperature can drop to minus 50 °C and lower, buildings must be insulated. In such buildings, effective slab insulation is used. Taking into account the specifics of the application, slabs based on foamed polymers are used: slabs of extruded polystyrene foam (XPS) or polyisocyanurate foam (PIR). The slabs are laid with a displacement of the seams (in one run) in 2–3 layers along the base and along the surface of the vertical walls. Nevertheless, cold bridges remain both along the contact surfaces between the panels and between the thermal insulation and the structural elements of the building. The outer surface of the structure facing the ground is not insulated, allowing cold air to enter through the cracks in the structure and cold air to enter through the material of the load-bearing elements (see Figure 8). The load-bearing structure itself is located in the cold zone, which negatively affects its durability.

**Figure 9:**
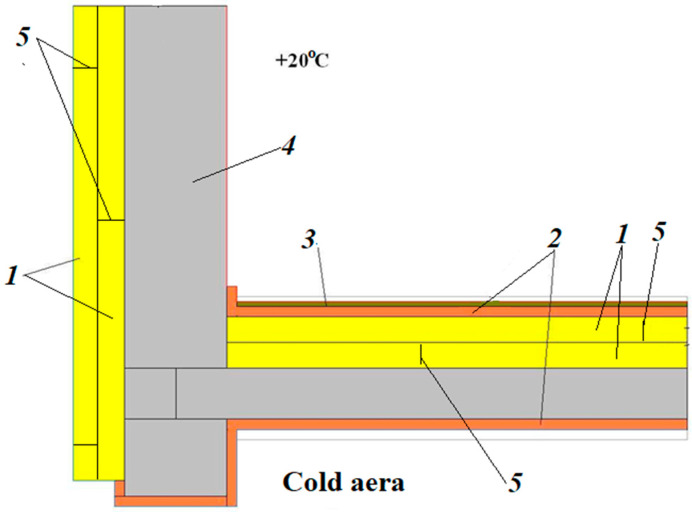
Insulation system with polyethylene foam and slab products made of extruded polystyrene foam (XPS) or polyisocyanurate foam (PIR): 1—slab thermal insulation (XPS or PIR); 2—rolled polyethylene foam; 3—chrysotile cement sheet; 4—load-bearing structural elements; 5—butt joints between slabs.

Rolled material with a reflective coating not only gives the structure additional stability and resistance in harsh climatic conditions, but also completely isolates the convective heat exchange zones at the joints (filtering and extracting cold air from the environment). Rolled polyethylene foam with a reflective coating (foil or metallized) also forms a protective layer along the outer perimeter of the structure and, on the inside, forms the basis for a floating floor laid on the panels of the supporting structure (Figure 9).

The polyethylene foam rolls are laid in place and fixed by mechanical fastening with wide-head plastic dowels. The interlock joint is formed during the installation of the panels, then the panels are welded, and a seamless insulation shell is formed. The outer protective cladding is made of non-combustible materials. Inside the room, the rolled polyethylene is laid and fixed with the dry construction method “floating floor”, where the rolled insulation must be placed at least 100 mm high on the wall before laying the substructure of the floating floor (fibre cement boards) and the top covering. The thickness of sheet thermal insulation (XPS or PIR) is 200 mm, and the thickness of roll insulation based on polyethylene foam is 50 mm.

**Figure 10:**
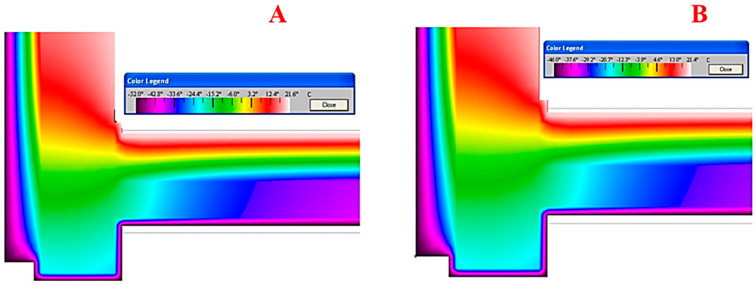
The structure of the formation of the temperature field in a structure isolated above the ventilated space (between the supporting columns): (**A**) visualization of the temperature field for Yakutsk (cold minus 52 °C); (**B**) visualization of the temperature field for Norilsk (cold minus 46 °C).

Structural solutions based on the use of a combined insulation system make it possible to form an insulation shell that meets the requirements of both heat saving and energy efficiency. The effect of this system solution increases when insulating structures that operate in conditions with significant subzero temperatures (Figure 10). Studies at NRU MGSU have shown that insulation materials based on polyethylene foam can be used at temperatures as low as minus 80 °C without losing their properties and thermal insulation capacity.

The modelling shows that where the structure rests on the column, there remains an area of undesirable heat transfer through the column structure and on to the truss and load-bearing elements of the building due to the high thermal conductivity of the load-bearing structures. A “temperature bridge” is formed, which passes through the points of contact of the structural elements: “column-timbered load-bearing wall”. As a result, the efficiency of the internal insulation system is somewhat reduced due to the fracture of the insulation shells at the supports of the columns (see Figure 8).

The distance between the columns is 4–6 m; therefore, such areas of increased heat transfer can be considered local. However, in order to increase the comfort of the microclimate parameters in the room, it is possible to apply a heat-insulating shell along the inner side of the walls.

The application of rolled polyethylene foam on an insulated external surface (above the cold space under the structure) and as an element of the floating floor inside the room makes it possible to block or minimize possible heat losses due to both the “temperature bridge” and the direct penetration of cold air into the construction joints.

All the advantages of polyethylene foam—not only its low thermal conductivity, but also its low vapour and water permeability, as well as its low water absorption—are used in systems to protect permafrost floors from thawing. Permafrost soils occupy significant areas both in Russia and in other countries bordering the polar zones (USA, Canada, Denmark, Norway, Sweden, etc.). When building in these regions, it is essential to take into account the need to maintain the soil in a frozen state. When such soil thaws, the bearing capacity of the subsoil is reduced to a minimum, and methane release and other undesirable effects are possible.

Thawing of frozen ground occurs due to the radiative effect of solar heat as well as heat loss through the foundations of poorly insulated buildings. A major threat to frozen ground is water (rain, melting).

**Figure 11:**
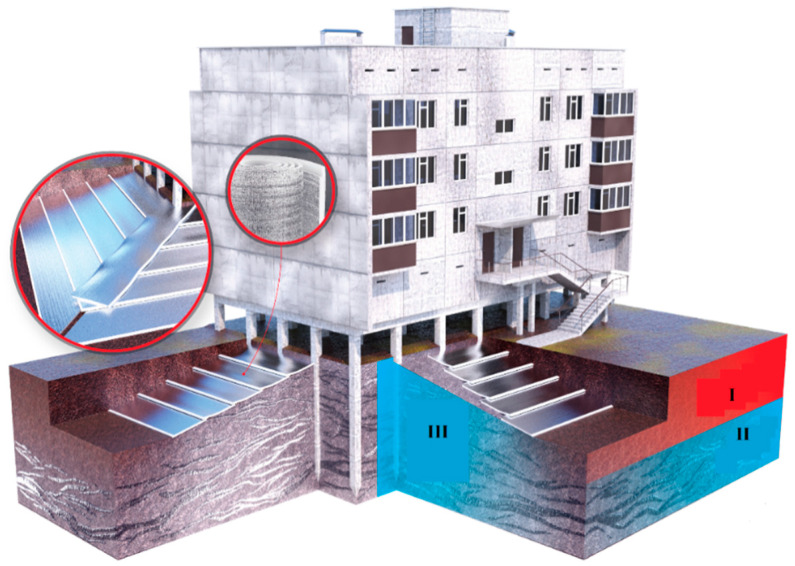
Protection of the base of the building (permafrost soil): I—layer of summer thawing; II—permafrost; III—permafrost persists throughout the depth.

Works on the protection of permafrost soil can be implemented in the following sequence (Figure 11). The soil is preliminarily removed along the perimeter of the building in a strip, the width of which is 1.5 m. The depth of excavation is 0.5 m. Rolled polyethylene foam (with a reflective coating on top) is placed in a pit with a slope of 3–7% and, if necessary, welded at the joints. A seamless insulation shell is formed along a horizontal surface (see Figure 1). The pit is then backfilled with excavated material. Next, the pit is filled with excavation soil. In this system, polyethylene foam, firstly, performs the function of cutting off and removing rain and melt water that forms near the building, and, secondly, is a heat insulator that prevents the penetration of external heat into the frozen ground.

## 4. Conclusions

Seamless shells based on rolled polyethylene foam have stable thermal performance, which is due to the minimization of cold bridges, the absence of leaky joints, the low thermal conductivity, vapour and water permeability of the base material and its low water absorption. These systems are used in the insulation of low-rise buildings, first floors, flat and pitched roofs in various climatic regions, preserving permafrost soils. The conducted studies have shown that it is possible to obtain a self-extinguishing modified polyethylene foam, as the material is very complex during its technological processing. The possibility of using a flame-retardant foaming agent masterbatch, which slightly affects the ability of the material to foam in the extruder, and the possibility of using clays of the montmorillonite group, as well as replacing the foaming agent with carbon dioxide, which does not support the combustion process, were studied. The optimum consumption of flame retardant was set at 3.7 … 3.8%; a nomogram and a computer program algorithm for predicting the properties of modified polyethylene foam were developed.

One promising direction is the use of jointless insulation systems in Arctic conditions. These systems can be used to create insulating envelopes for buildings on stilts and to insulate the ground to maintain its frozen state at positive air temperatures and prevent the effects of meltwater.

Reducing the flammability of polyethylene foam makes it possible to expand the scope of application of this material and increase the level of safety of building structures, both in terms of fire load and in terms of increasing reliability and durability, as well as ensuring the safety of logistics chains for the violation and delivery of this material. Materials for thermal insulation systems for buildings and structures in northern latitudes should have not only low thermal conductivity, but also high operational strength and stability of properties under difficult operating conditions. The calculation of temperature fields using the computer program THERM has shown that the developed insulation systems for building structures allow standard thermal resistance and minimize possible heat transfer bridges. The rolled polyethylene foam with a metallized coating, forming a seamless insulating shell, completely blocks the possible paths of cold air penetration at the joints of the load-bearing components, thus reducing possible heat losses.

## Figures and Tables

**Table 1:** Experiment conditions.

Factor Name	Symbol X_i_	Average Value of the Factor $\bar{X}$i	Variation Interval, ΔX_i_	Factor Values at Levels	
				−1	+1
Modifier content [%]	X_1_	4	1	3	5
Flame-retardant consumption [kg/m^3^]	X_2_	3	2	1	5
Extruder pressure, [kPa]	X_3_	90	10	80	100

**Table 2:** Validation of the obtained data according to the results of the active experiment.

№	Variable Parameters		Average Density [kg/m^3^]			Oxygen Index [%]		
			Meaning		Variance [%]	Meaning		Variance [%]
	Modifier Consumption [%]	Pressure in the Extruder [kPa]	Analytical	Experimental		Analytical	Experimental	
1	5.0	100	20.5	21.4	4.4	16.8	17.2	2.4
2	5.0	80	28.7	27.2	5.2	32.0	30.9	3.4
3	4.5	90	24.4	25.0	2.5	29.4	28.0	4.8
**4**	**3.5**	**100**	**31.7**	**30.1**	**1.9**	**32.4**	**31.0**	**4.3**
**5**	**3.5**	**80**	**23.3**	**24.4**	**4.7**	**35.6**	**34.1**	**4.2**
**6**	**3.0**	**90**	**33.0**	**31.9**	**3.3**	**33.0**	**32.0**	**3.0**
7	4.0	100	26.1	25.7	1.5	29.6	28.2	4.7
8	4.0	80	31.5	30.8	3.7	32.8	33.5	2.1
9	3.5	90	28.8	28.1	2.4	31.2	32.4	3.8

## Data Availability

Data confirming the reported results can be found on the website of Tepofol LLC.
